# Children's Trait Inference and Partner Choice in a Cooperative Game

**DOI:** 10.1111/cdev.14247

**Published:** 2025-04-12

**Authors:** Laura Schlingloff‐Nemecz, Maayan Stavans, Barbu Revencu, Kazuhide Hashiya, Hiromi Kobayashi, Gergely Csibra

**Affiliations:** ^1^ Department of Cognitive Science, Cognitive Development Center Central European University Vienna Austria; ^2^ TUM School of Social Sciences and Technology Technical University of Munich Munich Germany; ^3^ Cognitive Neuroimaging Unit, CEA, INSERM, NeuroSpin Center Université Paris‐Saclay Gif‐sur‐Yvette France; ^4^ Faculty of Human‐Environmental Studies Kyushu University Fukuoka Japan; ^5^ School of Psychological Sciences, Birkbeck University of London London UK

**Keywords:** cooperation, partner choice, trait attribution

## Abstract

A series of experiments conducted in Central Europe (Hungary, Austria) and East Asia (Japan) probed whether 5‐ to 10‐year‐old children (*n* = 436, 213 female) and adults (*n* = 71, 43 female; all data collected between July 2020 and May 2023) would infer traits and choose partners accordingly, in a novel touchscreen game. The participants observed third‐party actions and interactions of animated agents whose behavior varied in prosociality and skill, and subsequently selected whom to play with in potentially cooperative endeavors. Overall, the results indicate (1) that trait inference may not naturally follow from action understanding but relies on learning and experimental task framing, and (2) that by 7 years of age, children begin to capitalize on such inferences in partner choice.

The capacity to evaluate and choose prospective partners may have played a crucial role in the evolution of uniquely human forms of cooperation and morality. In an environment that affords opportunities for mutualistic interactions, it is beneficial to select good partners and be chosen by others, as working together for a common aim can yield benefits for all those involved. It has therefore been argued that humans have evolved psychological mechanisms to successfully navigate their social environment (Martin et al. 2019), dubbed “partner choice psychology,” which are thought to account for much of human moral cognition (Baumard et al. 2013).

Research into the development of partner choice in young children has only recently begun to directly probe their ability to identify and choose advantageous partners for cooperative interactions (Grueneisen et al. 2023; Hermes et al. 2016; Martin et al. 2022; Prétôt et al. 2020; Woodward et al. 2022). These studies suggest that young children can ascribe cooperation‐relevant character traits from a third‐party perspective, which in turn informs their partner choice decisions. However, it is yet unknown whether they do so (1) when they have to infer traits from behavioral observation rather than verbal vignettes; (2) when they choose partners on the basis of these attributed traits without explicit prompts; and (3) when their partner selection has real consequences for their reward rather than being purely hypothetical.

The present research project aimed to address these gaps. Using a novel tablet‐based research game, we investigated how children and adults would choose partners when they can play together with their chosen partner. In the game, the outcome for a participant depended partly on their partner's behavior. Participants first observed animated agents from a third‐party perspective. The behavior of these agents was guided by preset trait parameters specifying their prosociality (here: helpfulness) and competence (here: speed). Then, participants selected one of the previously observed agents and played together with their chosen partner. We conducted a series of experiments with children (*n* = 436) and adults (*n* = 71) in both Central Europe (Hungary, Austria) and East Asia (Japan). The aim was to investigate whether participants can identify differences between agents from observing their behavior without verbal prompts highlighting the concepts under investigation, whether they prefer interaction partners who are more helpful and/or skilled, and whether they systematically prioritize one trait over another. Our study varies both trait domains in the same context and assess their respective influence on children's partner choice.

## 1 Cooperation, Partner Choice, and Trait Attribution

In environments that provide opportunities for mutualistic collaboration (i.e., agents stand to gain from an interaction), it is beneficial for agents to cooperate. Partner choice can be a viable mechanism for stabilizing cooperation in such an environment and safeguarding it from exploitation through free riders (e.g., Barclay 2016; Baumard et al. 2013; Noë and Hammerstein 1994). Agents who cooperate can reap the benefits yielded by joint endeavors, and those who have a good reputation are more likely to be chosen in the future.

It has been hypothesized that due to the role of partner choice in the evolution of human cooperation, present‐day humans are equipped with an evolved, dedicated partner choice psychology (Baumard et al. 2013; Martin et al. 2019). This allows drawing inferences about cooperation‐relevant character traits from observing agents' behaviors in third‐party interactions. To do so, observers have to distinguish between situational contributions to an outcome (such as luck) from agent‐specific traits that are stable across variable circumstances and contribute causally to the agent's behavior (Delton and Robertson 2012).

The value of a partner is generally thought to depend on the partner's *willingness* to confer benefits to the self (e.g., generosity, fairness, loyalty, or helpfulness) and *ability* to do so (e.g., skills, intelligence, or access to resources) (Barclay 2013, 2016; Fiske et al. 2007). Research suggests that human adults attend to both of these domains and use them in partner choice decisions (Barclay and Willer 2007; Bliege Bird and Power 2015; Cottrell et al. 2007; Landy et al. 2016; Macfarlan and Lyle 2015; Sylwester and Roberts 2010). Some of these studies suggest that participants prioritize willingness to cooperate over the ability to do so (e.g., Bliege Bird and Power 2015; Cottrell et al. 2007; Delton and Robertson 2012; Eisenbruch and Roney 2017; Landy et al. 2016; Raihani and Barclay 2016). However, some cross‐cultural investigations point to the possibility that trait attribution may not operate the same way across human populations. For example, extant literature has focused on comparing trait reasoning and behavior explanation in subjects from Western (US) and Eastern (Asian) cultural contexts, generally finding that the latter perform fewer spontaneous trait inferences and rely less on traits and more on situational and relational causes for behavior (e.g., Choi et al. 1999; Lee et al. 2015; Na and Kitayama 2011; Shimizu et al. 2017), and that this difference emerges in childhood (e.g., Chen et al. 2016; Lockhart et al. 2008). Moreover, culture and socialization may influence which traits are considered relevant (Macfarlan and Lyle 2015; Smith and Apicella 2020a, 2020b). For instance, Smith and Apicella (2020b) report that Hadza hunter‐gatherer adults' relative prioritization of potential campmates' character traits (hunting skill, generosity) may undergo change as a result of influence from outside cultural institutions.

Developmental research, especially conducted in different cultural contexts, on whether young children prioritize either a partner's willingness or ability to provide benefits can offer important insights in this debate. There have been repeated calls to increase sample diversity in developmental psychology (e.g., Nielsen et al. 2017; Amir and McAuliffe 2020), as the majority of participants in this field are sampled from WEIRD populations (in particular, the United States). Testing children from different backgrounds using the same experimental procedures allows exploring common patterns as well as variability in the development of human cognition and behavior. This is especially pertinent when studying hypothesized human universals, that is, evolved features of our cognitive system that may emerge in a way that is largely unaffected by culture and socialization.

## 2 Trait Attribution and Partner Choice in Children

If partner choice psychology is part of humans' evolved social cognition, it may already emerge early in ontogeny. Research on the development of cooperative behaviors and the cognitive mechanisms underlying them supports the view that children track socially relevant behaviors and enter into collaborative interactions from a very young age (Kuhlmeier et al. 2014; Warneken 2018). However, the mechanisms underlying more sophisticated, uniquely human forms of cooperation and their respective developmental trajectories are still unclear. Distinct bodies of research have produced mixed evidence on whether the skills required for choosing interaction partners on the basis of inferred character traits are already possessed by young children. Specifically, it is unclear whether children draw conclusions from agents' past behaviors and use them to predict how they will behave toward them.

On the one hand, research on young children's reasoning about others' behavior in terms of stable character traits has yielded mixed results. Some studies suggest that this ability is limited in early childhood and develops gradually over the elementary school years, with more robust trait attribution emerging around 7–8 years of age (Alvarez et al. 2001; Boseovski and Lapan 2021; Kalish 2002; Kalish and Shiverick 2004; Livesley and Bromley 1973; Rholes and Ruble 1984; for a review, see Heyman 2009). A study by Liu, Gelman, and Wellman (Liu et al. 2007) suggested that preschoolers succeed at inferring traits from observed behaviors and generating behavioral predictions from trait labels, but struggle with behavior‐to‐behavior predictions (cf. Fitneva and Dunfield 2010; but see Surian et al. 2018).

On the other hand, recent findings of children's and infants' social selectivity after observing agents in interactions are taken as evidence that from early on, children can recognize and choose good cooperators. Preverbal infants were found to prefer prosocial over antisocial characters (e.g., Hamlin et al. 2007; Hamlin and Wynn 2011; but see Salvadori et al. 2015; Schlingloff et al. 2020). From the second year of life, young children preferentially engage with those who previously acted prosocially toward another person (e.g., Burns and Sommerville 2014; Lucca et al. 2018; Olson and Spelke 2008; Vaish et al. 2010; for a review, see Kuhlmeier et al. 2014). In making such social decisions, recent findings suggest that children also take agents' intentions into account (Jara‐Ettinger et al. 2015; Kishimoto et al. 2018; Martin et al. 2022).

These findings have been interpreted by some in terms of an early‐emerging partner choice psychology (Kuhlmeier et al. 2014; Martin et al. 2019; Van de Vondervoort and Hamlin 2016). Conversely, Warneken (2018) has pointed out that even if young children engage in third‐party social evaluation, they may not leverage these evaluations to guide their own cooperative decision‐making. The requisite abilities supporting the latter may instead undergo a protracted development and not all emerge simultaneously. Thus, the precise distinct mechanisms involved in different social reasoning tasks remain to be identified.

Some recent studies have more directly investigated children's choice of partners. This research has demonstrated that children as young as 3 years can, under some circumstances, track an agent's past behavior, for example, whether they possess a certain skill (Grueneisen et al. 2023; Hermes et al. 2016), or how they behave in a social scenario (Grueneisen et al. 2023; Martin et al. 2022; Prétôt et al. 2020; Woodward et al. 2022), and use this information to guide their partner choice decisions.

One limitation of previous studies is that children were generally asked to make hypothetical assessments about fictional characters, or could express affiliative behavior toward others, but were not choosing an actual partner for a cooperative endeavor (but see Droege and Stipek 1993; Feldman and Ruble 1988; Hermes et al. 2015, 2016; Prétôt et al. 2020), such that the stakes of choosing a bad partner were low. This may limit the ecological validity of these studies. For example, in Grueneisen et al. (2023), children were told about agents through illustrated vignettes and had to choose partners for a hypothetical task; in Woodward et al. (2022), children watched videos and were asked who they would want to play with.

Another limitation is that the studies often rely on verbal vignettes as stimuli and experimenter prompts for eliciting responses. This may affect their results, as the framing could highlight the socially relevant features of the situation and the concept under investigation (Fitneva and Dunfield 2010; Heyman and Gelman 1999). For example, if the experimenter asks a question like “Who is *daxier*?” this conveys that the agents possess the trait of being *daxy* and implies that there is a difference in *daxiness* between agents. In the study by Grueneisen et al. (2023), for instance, the experimenter provided children with detailed, explicit information about the agents that is directly relevant for the task domain for which partner choice is elicited. In many real‐world partner choice scenarios, people infer trait dimensions directly from behavior observation.

Moreover, if the behaviors or traits are highly desirable and normatively prescribed in children's social environment (e.g., it is good to share and help), studies in which an experimenter elicits a response could bias children toward responding in a norm‐consistent way due to reputational concerns (Engelmann et al. 2012; Rapp et al. 2019). For example, Woodward et al. (2022) asked children how nice the agents were prior to expressing a partner choice. In real‐world partner choice, it can be advantageous to interact with an agent who violates these norms (but see Dhaliwal et al. 2022) because they can provide benefits in a specific context (e.g., selecting a ruthless, aggressive lawyer to defend oneself).

## 3 Aims of the Present Project

With our study, we aimed to address these gaps in the literature. In collaboration with a software company, we devised an iPad application called Co‐Collectors, which can be used in research on partner choice as well as other aspects of social cognition. The app consists of a foraging game, in which computer‐animated agents as well as players themselves collect resources, either alone or together, and the player's payoff depends in part on the behavior of their partner. Here, children gather information about social partners by observing their actions, and have to infer agents' traits from observed behavior patterns—insofar as they interpret these patterns as indicating stable dispositions.

We focused on the character traits of prosociality and skill, which are often used in the literature as examples for a partner's willingness and ability to confer rewards to the agent. In our game, prosociality means helpfulness toward the partner, and skill is operationalized as speed, which results in higher foraging success. These traits can be varied continuously, such that agents can be relatively more or less prosocial or skilled. Because trait dimensions are orthogonal to one another, both the individual and the interaction effects of these traits can be investigated.

Our study targeted the age range around the onset of formal schooling, from late preschool through the elementary school years (i.e., 5‐ to 10‐year‐old children), as prior studies have found development in trait attribution during this time. We also collected data from adult participants, who we predicted would choose more advantageous partners based on inferred traits, and compared their responses to those of children.

We further wanted to explore whether there might be systematic differences in children's preferences that could be driven by cultural influences. We conducted experiments in both Central Europe (Hungary, Austria) as well as East Asia (Japan), using the same materials in both sites, with testing scripts translated into the respective languages. As mentioned before, these two cultural contexts (Western and East Asian) have been contrasted in research on trait attribution, suggesting that people from the latter may be less likely to spontaneously attribute traits. (We here take Central Europe to be part of the West, although most of the previously cited literature relies on North American samples, and it is possible that there are important differences in trait reasoning between the two regions.) We tested both adult and child participants in both settings and compared the responses of the age‐matched samples, respectively. This allowed us to explore (1) whether there are systematic differences in participants' partner choices, and (2) whether such differences already emerge in childhood.

The previous findings on cultural differences in trait attribution between people from Eastern and Western backgrounds permit different conjectures for our study. On the one hand, if partner choice psychology is an evolved, early‐emerging adaptation aimed at identifying partners who are helpful and competent, with a prioritization of the former, it is possible that the responses of the Japanese and Central European groups do not differ. On the other hand, if there is an influence of culture‐specific folk psychological beliefs (e.g., about the causes of actions) and norms and values guiding cooperative decision‐making, we may observe different patterns of results. Given our implicit measure and the multitude of potentially relevant dimensions, it is difficult to predict the precise nature of such differences. One possibility is that people who are less likely to explain behaviors in terms of traits are less inclined to choose advantageous partners based on third‐party observations. Another possibility is that there are systematic differences in the types of character traits that people assume to exist and be task‐relevant. Finally, explanations invoking other features than traits (e.g., effort and persistence rather than skill) may shape partner choice.

## 4 The Co‐Collectors Game

Our aim with the game was to investigate children's partner choice in a situation where potential partners' collaboration‐relevant traits must be inferred from observing their behavior (Goal 1); partner choice affects children's payoff in the task at hand, rather than being abstract or hypothetical (Goal 2); and children choose a partner without being prompted or supervised by an experimenter (Goal 3). We designed the game to be structured such that these goals are met.

**FIGURE 1:**
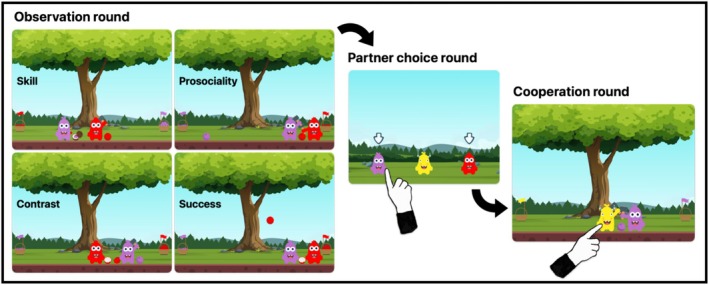
Illustration of the structure of a trial in the Co‐Collectors game (Experiments 2–4). Participants, who always played as a yellow kobo avatar, first watched a video clip of two agents collecting berries (Observation round, left; each trial contained only a single video clip of a particular type), then selected one of the two agents from the preceding video (Partner choice round, middle), and finally played together with the chosen partner (Cooperation round, right). Kobo colors varied across trials, and their identity was counterbalanced. Experiments 1–2 featured all four trial types, Experiment 3 only had Skill and Prosociality trials, and Experiment 4 had Skill, Prosociality, and Contrast trials. Note that in Experiment 1, participants did not play the game, but only watched videos of Observation rounds and responded to questions about them.

In the game, participants can both play themselves and watch other agents play (see Figure 1 for an overview of the game structure). After observing animated agents whose “personality” profiles vary in prosociality and skill (“Observation” rounds), the participant chooses which of the characters they want as a partner for a subsequent round (“Partner Choice” rounds) and then plays with that partner (“Cooperation” rounds). The participant has to infer who would make a better partner from the agents' previous behavior (Goal 1). How well the participant does depends partly on whether they choose the relatively more prosocial or more skilled partner (Goal 2). After an introduction phase, participants can play independently without supervision as the app automatically logs their choices (Goal 3).

In the game, players collect resources for themselves, but there are opportunities for mutualistic collaboration as well as for altruistic helping. The primary motivation should be to maximize one's own reward, and thus select partners that aid in the pursuit of this aim. This means that on the one hand, they should choose a highly skilled partner. Such a partner is advantageous even if they are not particularly altruistic, because in a mutualistic cooperative interaction they will generate windfall benefits for the partner even if otherwise pursuing a self‐interested strategy. On the other hand, participants should also choose agents who act prosocially. This is beneficial because when the collaborative option does not yield the highest payoff, choosing an altruistic partner who sacrifices their own resources on behalf of another is a good strategy. In the Supporting Information (Section B), we provide an overview of how many resources, on average, the agents in the Observation rounds obtained in our stimuli.

For a detailed description of the Co‐Collectors game, see Section A in the Supporting Information. In the game, small, colorful, monster‐like autonomous agents called “kobos” collect berries. The berries are contained inside “coconuts,” which fall from a tree and have to be cracked open by the kobos using little hammers, else they disappear. The coconuts are either colorful, matching in color a kobo in the scene, or brown. Colorful coconuts are “proprietary”: they contain berries that are awarded to the matching kobo, irrespective of who cracks them open. In contrast, brown coconuts are “shared”: they contain berries of two different colors, corresponding to the two kobos present. A skilled kobo is one who, all else being equal, is more successful at cracking. This is implemented by defining the speed at which an agent approaches coconuts and delivers hits. A helpful kobo is one who assists its partner in cracking its coconuts and forgoes its own rewards to do so. A unique feature of the game is that an agent's degrees of skill and prosociality—and the fact that these are relevant dimensions—must be inferred from the agent's behavior patterns. The observer thus cannot simply rely on verbal cues or familiar prototypical action features.

## 5 The Present Study

Our main aims were to investigate (1) whether children would prefer partners who are relatively more helpful and more skilled when playing the game by themselves, (2) whether they would systematically prioritize one trait over the other, (3) whether there would be differences between the choices made by the Central European and Japanese participants, and (4) whether adult participants would behave differently than children in the same task.

In Experiment 1, we tested whether 5‐ to 10‐year‐old children would recognize differences in the agents' behaviors. This served as a comparison baseline for Experiment 2, where children (and adults) played the Co‐Collectors game with the same stimuli, but now had to choose partners with whom to play. In Experiment 3, we slightly modified the experimental procedure to test children's choice of potential partners who varied in helpfulness or skill, but not both. Finally, in Experiment 4, we probed whether elementary school‐aged children would prioritize one of these traits when the two were in contrast. In Experiment 1, data were collected online; in Experiments 2–4, data collection took place in person, using the Co‐Collectors iPad app. Table 1 gives an overview of the designs used in the four experiments. All materials (stimuli, experimental scripts, data, statistical models, and their output) can be accessed at https://osf.io/p8uhj. Note that while the plans for data analysis within a sample were preregistered for all experiments, we did not specify from where samples would be drawn, nor did we preregister the direct comparisons between samples. In this sense, the analyses comparing Hungarian and Japanese participants (Experiments 1–3) and children and adults (Experiment 2) should be considered exploratory.

**TABLE 1:** Overview of the Experiments 1–4.

Experiment	Version	Country	Age	Venue	Task
1	A	Hungary	Children (5–10 years)	Zoom	Identify behavior differences between agents (speed, rate of helping)
	B	Japan			
2	A	Hungary	Children (5–10 years)	Live (iPad)	Choose interaction partner after observing agents who differ in skill and/or prosociality
	B	Hungary	Adults		
	C	Japan	Adults		
3	A	Hungary	Children (5–10 years)	Live (iPad)	Choose interaction partner after observing agents who differ in skill or prosociality (modified procedure)
	B	Japan			
4	—	Austria	Children (7–10 years)	Live (iPad)	Choose interaction partner after observing agents who differ in skill and/or prosociality (modified procedure)

## 6 Experiment 1

Before conducting the experiment with the iPad paradigm, we wanted to ensure that children can recognize the differences between kobos' rate of helping and speed. This served (1) to establish that they understand the relevant aspects of the stimuli and (2) as a comparison with the task in Experiment 2, where children chose partners from among the same kobos.

The experiment was preregistered (https://osf.io/abr98).

### 6.1 Methods

#### 6.1.1 Participants

Participants were 5‐ to 10‐year‐old children, recruited such that they spread approximately evenly across the age range (~10 per year cohort). In all experiments, caregivers gave consent for their child to participate (E1: verbally, E2–4: written), and children were asked whether they wanted to take part. The experiment, as well as all following ones, received full ethical approval from the respective university's research ethics committee. According to these ethical approvals, no additional socio‐demographic information was collected from participants.

##### 6.1.1.1 Experiment 1a (Hungary)

Sixty children participated in Experiment 1a (31 female, mean age: 7.8 year/93.9 month, SD: 20.1 month; data collected July–November 2020). Two additional participants were excluded due to caregiver intervention (*n* = 1) or experimenter error (*n* = 1). Participants were recruited via Facebook advertisement and received a gift voucher for their participation.

##### 6.1.1.2 Experiment 1b (Japan)

Sixty children in the same age range as Experiment 1a participated in Experiment 1b (30 female, mean age: 8 year/95.8 month, SD: 20.6 month; data collected June–August 2021). One additional participant was excluded due to dropping out early. Participants were recruited through the lab's database and received a gift voucher.

#### 6.1.2 Stimuli

The test stimuli were eight videos of kobos collecting berries. These trials were of four different types (two trials per type): Prosociality trials, in which agents differed in how much they helped the other (with an intermediate setting of the “skill” parameters); Skill trials, in which agents varied in how competent they were (with an intermediate setting of the “prosociality” parameters); Contrast trials, in which agents varied in both of these traits such that one was highly skilled but selfish and the other was helpful but incompetent (using the same high and low parameter values from the Prosociality and Skill trials, respectively); and Success trials, where agents did not differ in their traits (all parameter values were set to intermediate levels), but the distribution of resources was skewed in favor of one agent.

#### 6.1.3 Procedure

We ran the experiment online, using the Zoom video chat software for interacting with participants and Slides.com for presenting stimuli. The child first played a warm‐up game with the experimenter, where they were asked to identify colorful animal figures.

The experimenter then introduced the structure of the game; importantly, without explicit reference to agents' traits or differences. The experimenter asked several comprehension questions relating to the reward structure and contingencies of the game (see testing script on the OSF page). If a child answered incorrectly, the experimenter corrected them and repeated the question up to two times.

At this point, caregivers were asked to turn away from the screen. Children watched eight test trial videos and were asked a question after each one. Depending on the trial type, the question was “Which one helped the other more?” (Prosociality trials, one Contrast trial), “Which one was faster?” (Skill trials, the other Contrast trial), or “Which one collected more berries?” (Success trials). Trials were presented in pseudo‐random order, such that while watching a video, children did not know what question they would be asked afterward.

We counterbalanced trial order and agent identity (i.e., kobo color). To be included in the sample, children had to respond correctly to all comprehension questions after a maximum of three prompts, and provide a valid response to at least one trial per trial type. A child was excluded if they selected the agent on the same side across all trials.

#### 6.1.4 Coding and Analyses

Children's responses were recorded by the experimenter off‐line from the video recordings. For each of the samples, data 1/3 of the participants was recoded by an independent second coder to assess reliability; in both cases, there was 100% agreement between coders. We analyzed the data from this and all following experiments by fitting Bayesian logistic regression models to estimate the response parameter. This approach has the advantage that it allows describing the full posterior distribution of parameter estimates and avoids the issue of having to correct for multiple comparisons. As preregistered, we wanted to assess whether children correctly identified the target agent above chance. Specifically, we tested whether the proportion of correct responses varied by trial category (i.e., trials where only one of the agents' traits differed (Prosociality/Skill), both (Contrast), or only the outcome differed (Success)), whether children were more accurate in identifying who helped or who was faster, and whether the serial position of the trial mattered. In this and all following experiments, children's age, gender, and the counterbalanced stimuli group were added as predictors to rule out that they affected results. We report 89% credible intervals (CI) of parameter estimates for responding correctly (where 0 is incorrect, 1 is correct, and 0.5 is the chance level), or for the difference between trials or groups (where chance is excluded if the 89% CI does not include 0). These credible intervals specify that the true parameter value lies in this interval with 89% probability. We report primarily the findings that answer our main questions or where chance is excluded; for all results, see the model code and outputs on the OSF. The analyses were performed using R statistical software (R Core Team 2023), using the rethinking package (McElreath 2020).

### 6.2 Results

#### 6.2.1 Experiment 1a (Hungary)

**FIGURE 2:**
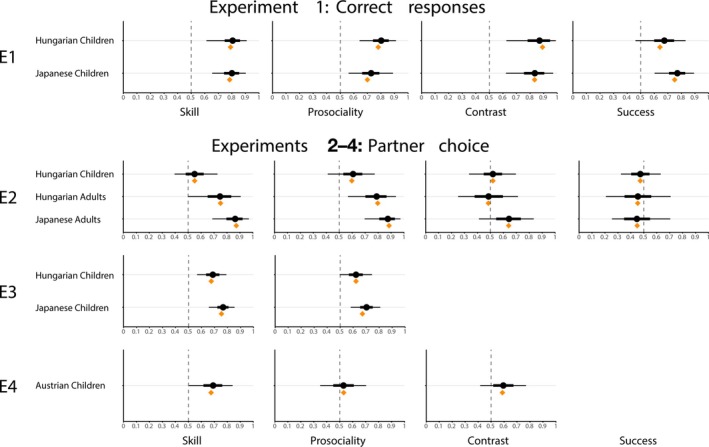
Overview of the results from Experiments 1 to 4 across the four trial types (Skill, Prosociality, Contrast, and Success). Orange rhombi indicate the observed proportion of a response in a trial. Black circles and lines indicate summary statistics of the posterior distributions generated by the Bayesian model (circle: mean of the distribution, thick lines: 89% credible interval, thin line: posterior range). On the *x*‐axes are the proportion of correct responses (Experiment 1) or proportion of choices for one of two agents (Experiments 2–4). In Contrast trials, choosing the more skilled agents is represented on the “correct” side (Experiments 2–4).

Hungarian children responded correctly above chance in all four trial types (Prosociality: *M* = 0.801, 89% CI: [0.741, 0.857]; Skill: *M* = 0.805, 89% CI: [0.747, 0.86]; Contrast: *M* = 0.876, 89% CI: [0.824, 0.924]; Success: *M* = 0.674, 89% CI: [0.599, 0.746]; see Figure 2). They were more accurate in Contrast trials, where both traits varied, compared to trials in which agents differed only in one trait (difference: *M* = 0.071, 89% CI: [0.012, 0.132]). Participants were more accurate in both of these trial categories compared to Success trials, in which only the distribution of resources varied (Contrast vs. Success: *M* = 0.202, 89% CI: [0.111, 0.294]; Prosociality/Skill vs. Success: *M* = 0.13, 89% CI: [0.051, 0.213]). They were equally likely to identify who helped more and who was faster, whether agents varied only in one trait dimension (*M* = −0.004, 89% CI: [−0.081, 0.071]) or in both, that is, Contrast trials, though here there was a tendency to be more accurate in recognizing speed differences (*M* = −0.069, 89% CI: [−0.154, 0.01]).

Hungarian children's accuracy tended to improve with age, though only in Prosociality trials did the age parameter estimates exclude chance (*M* = 0.462, 89% CI: [0.097, 0.834]; see Figure S3). We also examined whether they responded more accurately in later compared to earlier trials. A regression analysis indicated that the chance value for the accuracy increase was not excluded (*M* = 0.112, 89% CI: [−0.071, 0.293]). Thus, the accuracy was, on average, already above chance in the beginning (Figure S4).

#### 6.2.2 Experiment 1b (Japan)

Japanese children's responses were also correct above chance in all four trial types (Prosociality: *M* = 0.727, 89% CI: [0.661, 0.788]; Skill: *M* = 0.8, 89% CI: [0.742, 0.853]; Contrast: *M* = 0.838, 89% CI: [0.784, 0.887]; Success: *M* = 0.77, 89% CI: [0.709, 0.828]); see Figure 2. They were also more accurate in Contrast trials compared to those where only one trait varied (*M* = 0.071, 89% CI: [0.009, 0.134]). Unlike the Hungarian children, Japanese children's accuracy in the Success trials was similar to that of other trial types (Contrast vs. Success: *M* = 0.068, 89% CI: [−0.006, 0.145]; Prosociality/Skill vs. Success: *M* = −0.004, 89% CI: [−0.069, 0.066]). Hungarian children were less accurate in Success trials than their Japanese peers (*M* = −0.096, 89% CI: [−0.189, −0.007]). In no other trial did responses differ between the groups.

Japanese children's accuracy increased with age only in Skill trials (*M* = 0.35, 89% CI: [0.004, 0.7]) and for the helping question in Contrast trials (*M* = 0.475, 89% CI: [0.015, 0.942]; see Figure S3). The accuracy improved over the course of the experiment (*M* = 0.244, 89% CI: [0.058, 0.428]), though here, too, responses were already above chance in the first trial (Figure S4).

### 6.3 Discussion

The results from Experiment 1 provided evidence that children in our target age group recognize differences in agents' behaviors along the predetermined trait‐relevant dimensions in our stimuli. Even at a young age, from 5 years, and already from the first test trial on, participants attended to these behaviors without being cued about them in advance. We found that Hungarian children's responses were the least accurate in the Success trials, which suggests that they did not solve the task by merely attending to outcomes. In contrast, Japanese children were relatively more accurate in Success trials, suggesting that they paid relatively more attention to differences in reward that were not driven by agents' behaviors. This pattern, while not predicted by us, may point to an early‐emerging difference in how children from the two backgrounds attend to events.

Importantly, we asked children about behaviors rather than traits. Concluding that behavior differences were caused by variation in underlying traits requires an additional inferential step, which is needed for partner choice because only stable traits could mediate between observed past and expected future behaviors. We turn to the questions of whether children attribute traits to kobos and choose partners on this basis in the following experiments.

## 7 Experiment 2

In the next step, we aimed to test whether children and adults would use the information obtained by observing kobos in a third‐party context to infer context‐invariant traits and guide their partner choice when playing the Co‐Collectors game. Participants watched the same videos as in Experiment 1, which allowed us to assess whether their understanding of agents' behavioral differences would give rise to corresponding partner preferences.

The experiment was preregistered (https://osf.io/y5sbm).

### 7.1 Methods

#### 7.1.1 Participants

##### 7.1.1.1 Experiment 2a (Hungary)

Sixty‐five children took part in Experiment 2a (28 female). Participants' age range, and the approximate distribution across this range, were the same as in Experiment 1 (mean age: 7.9 year/94.7 month, SD: 21 month; data collected September 2020–August 2021). An additional 13 children were excluded for failing the comprehension questions (*n* = 2), inattentiveness (*n* = 2), dropping out early (*n* = 4), experimenter error (*n* = 4), or technical failure (*n* = 1). Participants were recruited through various means: by approaching visitors at the Budapest Zoo, through information sheets given out at preschools, and through the lab's participant database. They received stickers for their participation.

##### 7.1.1.2 Experiment 2b (Hungary)

Thirty‐two adults took part in the experiment (22 female, mean age: 29.0 year, age range: 20–42 year, SD: 6.2 year; data collected September–November 2021). Participants were recruited through the University's research participation system and received vouchers for participation. All tested participants met the inclusion criteria.

##### 7.1.1.3 Experiment 2c (Japan)

Thirty‐nine adults took part in the experiment (21 female, mean age: 25.1 year, age range: 19–39 year, SD: 5.2 year; data collected June–August 2022). One additional participant was excluded for displaying a side bias. Participants were recruited on the University campus or through the lab's participant database and received a gift voucher.

#### 7.1.2 Stimuli

The stimuli were largely the same as in Experiment 1. In the introduction phase, participants additionally encountered trials where they played the game themselves. After choosing an avatar, they first played by themselves in a Practice round, then went through a brief version of an experimental trial sequence (Observation, Partner Choice, and Cooperation round). Having been familiarized with this structure, participants could then play the game independently. The test phase featured the same videos used in Experiment 1 as Observation rounds. Each clip was followed by a Partner Choice round (Figure 1) and a Cooperation round.

#### 7.1.3 Procedure

This experiment was conducted in‐person, using an iPad. Data were collected at different locations: at the CEU Cognitive Development Center's Child Lab at the Budapest Zoo and at various preschools in Budapest (Experiment 2a), and University laboratories (Experiments 2a–c). The experimenter introduced the game using the same script and comprehension questions as in Experiment 1, with an added practice phase. After reminding participants of the test trial structure, the experimenter told them that they would now play by themselves, then turned away and pretended to work. In Experiment 2a, accompanying caregivers were asked not to intervene, and, in the test phase, to fill out some paperwork so that they would not supervise children's gameplay. The order of stimuli, counterbalancing of factors (order and kobo identity), and exclusion criteria were the same as in Experiment 1.

#### 7.1.4 Coding and Analyses

Participants' partner choices were automatically logged by the game. The analysis was similar to that in Experiment 1: Now we assessed the probability of participants choosing a specific partner; in particular, one who was more prosocial and more skilled (note that, for comparison, choosing the skilled partner was marked as “correct” in Contrast trials). We tested whether participants had a relatively stronger preference for prosocial or skilled partners, whether their preference was more pronounced in trials where agents varied only in one trait (Prosociality, Skill) compared to both (Contrast), and whether responses differed as a function of a trial's serial position. Additionally, we compared the data from Hungarian children with those of Hungarian adults (Experiments 2a and 2b), and Hungarian adults with Japanese adults (Experiments 2b and 2c).

### 7.2 Results

#### 7.2.1 Experiment 2a (Hungary)

The only above‐chance preference children had was that for a helpful kobo in Prosociality trials (*M* = 0.607, 89% CI: [0.531, 0.678]); in the other trials, choices did not differ from chance. A direct comparison of children's responses in Prosociality with those in Skill trials showed that the estimates for the difference between the two did not exclude chance (*M* = 0.057, 89% CI: [−0.047, 0.16]), and neither in the comparison with Contrast trials (*M* = 0.088, 89% CI: [−0.018, 0.191]). Children's preference for helpful partners increased with age: Older children were more likely to pick the prosocial kobo (*M* = 0.715, 89% CI: [0.418, 1.027]). In other trials, there was no substantial change in choice patterns with age (see Figure S5). When comparing the choices children made in the first versus the second trial of a type they encountered, their preference for a helpful partner in Prosociality trials increased over time (*M* = 0.143, 89% CI: [0.009, 0.277]); in Skill trials, chance was barely not excluded for this comparison (*M* = 0.111, 89% CI: [−0.023, 0.246]).

#### 7.2.2 Experiment 2b (Hungary)

Hungarian adults preferred a more competent partner in Skill trials (*M* = 0.746, 89% CI: [0.651, 0.833]) and a more helpful one in Prosociality trials (*M* = 0.789, 89% CI: [0.702, 0.865]). They did not show a preference in Success trials (*M* = 0.454, 89% CI: [0.351, 0.557]) or in Contrast trials (*M* = 0.486, 89% CI: [0.378, 0.596]). The strength of partner preference in Prosociality and Skill trials did not differ (*M* = 0.042, 89% CI: [−0.088, 0.174]).

In comparison to the choices of children in Experiment 2a, adults' proclivities to choose the better partner were relatively more pronounced in both Prosociality and Skill trials (Prosociality: *M* = 0.191, 89% CI: [0.067, 0.308]; Skill: *M* = 0.203, 89% CI: [0.073, 0.327]).

#### 7.2.3 Experiment 2c (Japan)

Adult participants in Japan, like those in Hungary, preferred a helpful partner in Prosociality trials (*M* = 0.875, 89% CI: [0.809, 0.929]) and a skilled partner in Skill trials (*M* = 0.863, 89% CI: [0.796, 0.92]), and the relative strength of these preferences did not differ (*M* = 0.012, 89% CI: [−0.077, 0.102]). They did not show a preference in Success trials (*M* = 0.447, 89% CI: [0.348, 0.548]). However, in Contrast trials, unlike the Hungarian sample, Japanese participants preferred the skilled, selfish kobo over the incompetent, helpful one (*M* = 0.643, 89% CI: [0.545, 0.735]). This preference was less strong than that in Skill trials (*M* = −0.22, 89% CI: [−0.328, −0.114]) and that in Prosociality trials (*M* = −0.232, 89% CI: [−0.351, −0.117]).

When comparing the Hungarian and Japanese groups' responses directly, participants in Japan were relatively more likely to choose a skilled agent in both Skill (*M* = 0.117, 89% CI: [0.01, 0.225]) and Contrast trials (*M* = 0.158, 89% CI: [0.016, 0.302]).

### 7.3 Discussion

Adult participants in both Hungary and Japan chose partners as we predicted: they preferred a better partner in both Prosociality and Skill trials. However, they did not perform at ceiling, suggesting that the task was not trivial for them or that they may have explored different strategies. A main difference between the two groups was that the choice of skilled partners was relatively more pronounced in the Japanese sample. In fact, they selected a highly skilled but selfish partner over one who helped but was less competent, while Hungarians did not have a systematic preference in Contrast trials.

Hungarian children tended to somewhat prefer a more helpful partner over a selfish partner after observing the two agents in Prosociality trials, and this tendency increased with age (consistent with the developmental pattern in Experiment 1a). They did not have a preference in Success trials, which, together with the result from Experiment 1, suggests that mere difference in outcome was not particularly salient for children and did not constitute sufficient grounds to generate a partner preference. Children did not prefer either agent in Contrast trials, suggesting that they did not prioritize one trait over the other. However, contrary to what we predicted, participants did not select a competent partner in Skill trials, despite being highly accurate at detecting this difference between agents in Experiment 1.

There are multiple explanations for the pattern of results we obtained with children in Experiment 2a. One is that while children recognized behavior differences, they did not spontaneously attribute this variation to underlying traits of the agents. Because of this, they could not have generated an expectation for how the agents would behave in the future. In other words, children may have failed at behavior‐to‐trait inference. Another option is that children failed to generate trait‐to‐behavior predictions and did not use the information about agent differences gleaned from the stimuli in order to inform partner choice. Perhaps children inferred traits and even engaged in sociomoral evaluation of the helpful agent, but did not understand how these traits would affect their own performance in the game. Studies by Woodward et al. (2022) and Hwang and Markson (2020) recently found that sensitivity to third‐party behaviors and sociomoral evaluation on the one hand, and partner choice on the other, can diverge. A similar divergence may have played a role in our task.

Relatedly, it is also possible that at least some children interpreted the foraging task in the game as a competitive one. Some participants commented on how they wanted to “beat” their partner or “get more” than the other kobo. In this case, they may have chosen a partner who did not appear to pose a threat (i.e., the helpful kobo) and avoided the skilled kobo, who would prove to be a more difficult competitor (Grueneisen et al. 2023; Magid et al. 2018).

A final possibility is that the task was too demanding for children: In Experiment 2a, children had to not only comprehend the structure of the game and attend to agents' behaviors, but had to learn how to play the game and infer in what ways the variabilities in agents' behaviors were relevant for themselves. This, in combination with the complexity of the experimental structure (four different trial types, two per type, presented in pseudo‐random order), may have hindered children's performance. In fact, we found that the proportion of children's choices for a better partner in Prosociality and Skill trials increased in the second trial they encountered (Prosociality: from 52.3% to 67.2%, Skill: from 49.2% to 61%). This suggests that children might have learned to choose a better partner from the experience of playing the game. We attempted to address these potential shortcomings in Experiment 3.

## 8 Experiment 3

Experiment 3 was similar to Experiment 2a, but we implemented some key changes that were meant to address the issues laid out in the discussion section of Experiment 2.

The task was simplified in a number of ways: First, children received four trials each of only two trial types (Prosociality and Skill). The trials of the same type were presented together in blocks, which made it more predictable to participants which features of the stimuli to attend to. Moreover, instead of featuring all types of coconuts, Skill trials now had only brown and Prosociality trials only colorful coconuts. This way, children did not have to attend to all coconut types at the same time. In Skill trials, brown coconuts (which agents cracked together) were sufficient to show skill differences. In Prosociality trials, helpfulness was indicated by whether an agent only cracked its own or assisted with the partner's coconuts.

Further, to discourage children's interpretation of the game as competitive, we aimed to convey that cooperating (i.e., hitting a nut together) is more likely to lead to successful cracking than working alone. Children therefore participated in two additional Practice rounds during the introduction: one in which they acted alone and another in which they played together with a preset partner and where they collected more resources (enhanced by setting the coconut hardness parameter to a lower level). Crucially, we did not want to highlight the possibility of altruistic or selfish behavior, or that agents could differ along this trait dimension. Therefore, these trials only featured shared brown coconuts.

The experiment was preregistered (https://osf.io/7pf8u).

### 8.1 Methods

#### 8.1.1 Participants

Children of the same age range as in Experiments 1 and 2a were tested. The aim was to test a minimum of 60 children in each group, such that they were approximately evenly distributed across the age bins.

##### 8.1.1.1 Experiment 3a (Hungary)

Sixty children participated in Experiment 3a (23 female, mean age: 7.8 year/93.6 month, SD: 18.9 month; data collected May–August 2022). An additional 3 participants were excluded from the sample for displaying a side bias (*n* = 1), technical failure (*n* = 1), or quitting the game early (*n* = 1). Participants were recruited from the lab's database. They received stickers for their participation.

##### 8.1.1.2 Experiment 3b (Japan)

Seventy children participated in Experiment 3b (28 female, mean age: 7.7 year/92.2 month, SD: 21.2 month; data collected April–June 2023). An additional 5 participants were excluded for interference by a parent or sibling (*n* = 2), dropping out early (*n* = 1), experimenter error (*n* = 1), and color blindness (*n* = 1). Participants were recruited through the lab's participant database. They received a gift voucher for their participation.

#### 8.1.2 Stimuli

As in Experiment 2a, children received eight test trials. Now there were trials of only two types (four each): Prosociality and Skill trials, grouped in blocks. The trials in the Prosociality block only contained colorful coconuts, and the trials in the Skill block only contained brown coconuts. In comparison to the stimuli of Experiments 1 and 2, we set the trait parameters of the agents to more extreme values to make the differences between them even more apparent.

#### 8.1.3 Procedure

Data collection took place in the respective University's laboratory. The experimental procedure was similar to that of Experiment 2a, aside from a few modifications. First, each of the two blocks (a “Skill” block and a “Prosociality” block) was preceded by an introduction phase acquainting participants with the coconut type featured in the subsequent stimuli. A general introduction to the game was presented to all participants at the very beginning of the experiment, which included three additional training rounds to clarify that the game was not competitive. Children were told that the amount of stickers they would receive at the end of the experiment would depend on their success in the game. This was also meant to incentivize participants more strongly to pursue a benefit‐maximizing rather than a contrastive (competitive) strategy. After the testing phase proper, we collected another measure where the experimenter asked children explicitly about what type of partner would be more advantageous; see Section C, Supporting Information.

The order of blocks (Skill first or Prosociality first) and the identity of the kobos within trials were counterbalanced across participants. To be included in the analysis, participants had to contribute at least two trials of each type. Further, if a child failed to respond correctly to the comprehension questions preceding the two blocks after a maximum of three prompts, or chose the kobo on the same side across all trials, this child's data were excluded.

#### 8.1.4 Coding and Analyses

The logging of responses was the same as in Experiment 2. As before, we analyzed the data with a Bayesian logistic regression to estimate the probability of children choosing a more helpful and skilled partner. We tested whether either of these preferences was relatively more pronounced. Moreover, we assessed whether there was an order effect: specifically, whether there was an effect of block and an interaction of block with trial type, and whether choices changed across the four trials within a block. Finally, we tested whether there were differences between the groups of Hungarian and Japanese children.

### 8.2 Results

#### 8.2.1 Experiment 3a (Hungary)

Hungarian children chose the more helpful partner in Prosociality trials (*M* = 0.622, 89% CI: [0.567, 0.674]) and the more skilled partner in Skill trials (*M* = 0.687, 89% CI: [0.635, 0.738]) above chance. There was no difference between the strengths of these preferences (*M* = −0.065, 89% CI: [−0.139, 0.009]). In both trial types, the tendency to choose a better partner increased with age (Prosociality: *M* = 0.497, 89% CI: [0.259, 0.738]; Skill: *M* = 0.766, 89% CI: [0.511, 1.031]; see Figure S6), such that older children from around 7 years were more likely to pick the more helpful and skilled partner.

There was an effect of block, such that the tendency to choose the better partner was higher in the second compared to the first block (*M* = 0.096, 89% CI: [0.025, 0.172]; see Figure S7). In particular, children who encountered Prosociality trials in the first block subsequently chose the more competent partner at a higher rate in the Skill trials of the second block (*M* = 0.151, 89% CI: [0.051, 0.26]); for children who first encountered Skill and then Prosociality trials, this effect did not exclude chance (*M* = 0.041, 89% CI: [−0.059, 0.144]). There was no interaction with the trial type, suggesting the increase from the first to the second block was similar for both trial types (*M* = −0.02, 89% CI: [−0.36, −0.311]). Moreover, children's choice of the better partner increased across the four trials within a block (Prosociality: *M* = 0.391, 89% CI: [0.128, 0.657]; Skill: *M* = 0.475, 89% CI: [0.203, 0.749]).

#### 8.2.2 Experiment 3b (Japan)

Just like their Hungarian peers, Japanese children preferred helpful and skilled partners above chance (Prosociality: *M* = 0.703, 89% CI: [0.654, 0.751]; Skill: *M* = 0.766, 89% CI: [0.722, 0.808]). The comparison between preferences in the two trial types did not exclude chance, though just barely: Children were somewhat more likely to choose a skilled partner (*M* = −0.063, 89% CI: [−0.129, 0.003]).

Here, too, older children were more likely to select helpful and skilled partners (Prosociality: *M* = 0.818, 89% CI: [0.589, 1.052]; Skill: *M* = 0.452, 89% CI: [0.232, 682]; see Figure S6). The order effects mirrored closely those found with Hungarian children in Experiment 3a. There was also an effect of block (*M* = 0.118, 89% CI: [0.051, 0.192]; Figure S7), similarly driven by those participants who encountered Prosociality trials in the first and Skill trials in the second block (*M* = 0.208, 89% CI: [0.103, 0.325]; Skill‐first participants: *M* = 0.028, 89% CI: [−0.053, 0.112]). There was no interaction with the trial type (*M* = −0.023, 89% CI: [−0.31, −0.247]). In contrast to the Hungarian sample, Japanese children's choice of the better partner did not increase across the four trials within a block for either trial type (Prosociality: *M* = 0.075, 89% CI: [−0.176, 0.323]; Skill: *M* = 0.187, 89% CI: [−0.082, 0.458]).

Finally, compared to the Hungarian sample, the preference of Japanese children was relatively more pronounced in both Skill and Prosociality trials (Prosociality: *M* = 0.081, 89% CI: [0.009, 0.153]; Skill: *M* = 0.079, 89% CI: [0.014, 0.146]).

### 8.3 Discussion

In Experiment 3, children from around 7 years responded as we predicted, choosing a partner that would help them maximize their reward, both by helping and by being a skilled collaborator. This is approximately the age at which, according to prior research, more robust abilities to reason about traits and generate predictions from past behaviors emerge (Fitneva and Dunfield 2010; Kalish 2002; Rholes and Ruble 1984). This result differed from that of Experiment 2a, where children had a slight preference for a helpful but not a more skilled collaborator. The multiple changes to the stimuli and experimental procedure we made may have contributed to this outcome to varying degrees.

The results with Japanese children replicated the finding from Hungary that participants chose partners who were relatively more likely to help them and were more competent. In fact, both preferences were stronger than in Hungarian children. Therefore, in addition to the higher proclivity to select more competent partners, which we already found in Japanese compared to Hungarian adults in Experiments 2b and 2c, Hungarian and Japanese children also differed in how much they favored a helpful kobo over a selfish one.

In both groups, partner choices improved as children gained practice with the game. In particular, those who initially observed and interacted with agents differing in helpfulness were more likely to choose skilled partners in the subsequent block, despite having to attend to a different trait dimension and novel resource types (colorful/brown coconuts). This result may indicate that children learned in the first block that agents in this context possess character traits that remain stable across partners and generalized this knowledge when agents varied along another trait dimension. Participants who first participated in the Skill block did not show a similar improvement, which could suggest that children have different prior expectations about types of traits: the fact that agents consistently differ in skill may not warrant the inference that prosociality is a context‐independent, causally efficacious natural kind in this game.

## 9 Experiment 4

After finding that children, as predicted, preferred helpful and skilled cooperation partners in Experiment 3 (at least from a certain age), we returned to the question of whether they would prioritize either trait when the two are in contrast. In Experiment 2, neither children nor Hungarian adults had shown such a preference, while Japanese adults chose a skilled but selfish partner over a helpful but incompetent one. We applied the changes made in Experiment 3 to investigate children's behaviors in Contrast trials in a further experiment.

We also made some additional modifications to the experimental design. First, we tested only children between the ages of 7 and 10, as this was the age range in which children chose a better partner in Experiment 3. Second, Experiment 4 had a between‐subject design, such that each participant only received four trials of the same type (the same number as in Experiment 3; in a within‐subject design, including all three trial types would have made the procedure too long). Third, to present the same introduction phase to all participants regardless of condition, we included all coconut types in all stimuli, as in Experiments 1 and 2. Finally, we shortened the introduction phase and removed the comprehension questions, as children of this age did not struggle with understanding the instructions in the previous experiments.

The experiment was preregistered (https://osf.io/9xt6y).

### 9.1 Methods

#### 9.1.1 Participants

One hundred twenty‐one 7‐ to 10‐year‐old children participated in Experiment 4 (63 female, mean age: 9 year/107.8 month, SD: 13.5 month; data collected February–May 2023). An additional 14 participants were excluded for displaying a side bias (*n* = 8), technical failure (*n* = 3), parental interference (*n* = 1), color blindness (*n* = 1), and a language barrier preventing communication (*n* = 1). Participants were children of varying nationalities who were recruited at the Natural History Museum Vienna (Austria). They received stickers for their participation.

#### 9.1.2 Stimuli

Participants were assigned to one of three conditions. In each condition, children received four test trials of a single type: Prosociality, Skill, or Contrast trials. Trials in all conditions included all types of coconuts (brown and colorful). The trait parameters of the agents were the same as in Experiment 3; in Contrast trials, they were crossed with each other.

#### 9.1.3 Procedure

Data collection took place at the Natural History Museum Vienna. The procedure was similar to that of Experiment 3, with more concise instructions and no comprehension questions. The identity of the kobos was counterbalanced. To be included, participants had to contribute a minimum of two trials. If a child chose the agent on the same side on all trials, or if there was interference from another person, this child's data were excluded from the analysis.

#### 9.1.4 Coding and Analyses

The logging of responses was the same as in Experiments 2 and 3. Using a similar model as before, we tested whether children chose partners who were relatively more helpful and/or skilled, whether their preference for helpful partners in the Prosociality condition differed from that for fast partners in the Skill condition, and whether the tendencies to choose partners in these conditions differed from that in the Contrast condition. We also assessed whether responses differed as a function of the serial position of the trial.

### 9.2 Results

Participants chose the more skilled partner in the Skill condition (*M* = 0.691, 89% CI: [0.616, 0.761]). However, the rate of choosing a helpful partner in the Prosociality condition did not exclude chance (*M* = 0.529, 89% CI: [0.45, 0.608]). When comparing the two conditions directly, the preference was stronger in the Skill than in the Prosociality condition (*M* = 0.162, 89% CI: [0.052, 0.271]). In the Contrast condition, children chose the more skilled, selfish over a helpful, less skilled kobo as a partner above chance (*M* = 0.595, 89% CI: [0.517, 0.673]). The preference for a highly skilled partner did not differ between the Skill and the Contrast conditions (*M* = −0.065, 89% CI: [−0.177, 0.045]), while the preference for a helpful partner was higher in the Prosociality condition compared to the Contrast condition (*M* = 0.123, 89% CI: [0.011, 0.233]). We found an effect of participants' age only for children in the Skill condition, such that older children were more likely than younger ones to prefer a competent agent (*M* = 0.443, 89% CI: [0.129, 0.763], see Figure S8).

Finally, we found that for children in the Prosociality condition, the proportion of choosing a helpful partner increased across the four test trials (*M* = 0.457, 89% CI: [0.156, 0.767]), but not in the other conditions (Skill: *M* = 0.117, 89% CI: [−0.195, 0.437]; Contrast: *M* = −0.159, 89% CI: [−0.459, 0.136]; Figure S9).

### 9.3 Discussion

In Experiment 4, children preferred to play with a more skilled agent, and even prioritized a partner's skill over prosociality when the two were in contrast. However, they did not choose a relatively more helpful agent over a selfish agent. This finding differs from those in Experiment 2a, where children only showed a preference in Prosociality trials and were at chance in Skill and Contrast trials, and Experiment 3a, where children selected both helpful and skilled partners.

This pattern of results need not constitute a discrepancy. In Experiment 2, as discussed above, children might not have interpreted the task as we intended (e.g., they may have evaluated the helpful agent's behavior without ascribing a prosocial trait). In contrast, in Experiments 3 and 4, children received multiple subsequent trials of the same type, which may have helped them recognize that agents behaved consistently across trials, and that collaborating with certain partners yielded higher rewards.

If such an explanation is on the right track, why did children choose a helper above chance in Experiment 3 but not in Experiment 4? First, children's preference for helpful partners in Experiment 4 increased across the four test trials. Thus, if children had received even more practice with a second block of trials, their average preference may have been above chance too. Moreover, the task in Experiment 3 was simpler in one potentially crucial aspect: Here children only encountered proprietary colorful coconuts in Prosociality trials, whereas in Experiment 4, they faced all coconut types from the beginning. Learning about an agent's skill may have been easier for children, as every instance of hitting any coconut provides evidence about its speed, whereas inferring an agent's prosociality requires interpreting a pattern of coconut‐approach and ‐avoidance behaviors. Note, however, that children in Experiment 1, which also featured all coconut types, recognized which agent helped more with high accuracy. This discrepancy between Experiments 1 and 4 may be because in Experiment 4 children had to go beyond interpreting past behavior and infer a trait which would exert stable influence over agents' behaviors across settings and partners. The fact that children's choices of a helpful partner (but not those of a skilled one) increased over time supports the idea that such learning played a crucial role here. While children quickly recognized which agent helped more, their preference for such an agent as a partner depended on abstracting, across multiple repeated trials, that those who helped a third party would also behave helpfully toward them.

## 10 General Discussion and Outlook

The aim of the present project was to study children's and adults' trait attribution and partner choice in a context where (1) prospective partners' traits had to be inferred from behavioral observations, (2) the selection of an interaction partner had tangible consequences on participants' reward, and (3) responses were not elicited through an experimenter prompt. We conducted a series of experiments with 5‐ to 10‐year‐old children and adults in two different cultural settings (Central Europe and East Asia), using the Co‐Collectors game, a custom research tool. In the game, participants had to infer prosociality and skill from observing the behavior of agents in a novel context, conclude that these are persistent traits, and use this information to select a partner for a subsequent cooperative interaction. This partner choice affected their success in the game. Participants' choices were not made in response to an experimenter prompt, and we avoided explicitly referring to the concepts in question, to probe children's partner choice solely on the basis of behavior observation.

Children across our age range succeeded at identifying which agents were faster and helped more (Experiment 1). However, when provided with the same introduction and tested with the same stimuli in a task where they had to play the game and choose partners, children did not respond as we predicted: They preferred a more helpful partner slightly above chance, but not a more skilled one (Experiment 2a). One possible explanation for these results is that children interpreted the task as competitive; subtle changes in their motivation or goal can affect children's strategy in a game (Goddu et al. 2021; Rule et al. 2023). Another possibility is that children did not spontaneously explain the differences in behavior among agents in terms of stable character traits or that they failed to use this information to strategically select partners. Instead, they may have preferred the prosocial character due to an affective tagging mechanism that leads to general valence‐based evaluation (Dunfield et al. 2023).

In a modified procedure, where we simplified the task, presented children with blocks of trials of the same type, and highlighted the collaborative structure of the game, children succeeded: From around 7 years, they chose helpful and skilled partners (Experiment 3). In a between‐subject design where children only received half as many trials, they still preferred skilled partners, but did not select helpful partners above chance (Experiment 4). They also chose a skilled, selfish agent over a helpful, slow one (Experiment 4). This finding is contrary to proposals which have argued that prosociality is generally prioritized over competence in partner choice (e.g., Bliege Bird and Power 2015; Eisenbruch and Roney 2017; Raihani and Barclay 2016) and indicates that children may reason differently about different trait categories.

In Experiments 3 and 4, the opportunity to encounter agents differing along the same domain(s) on multiple successive trials provided information that can support a crucial inferential leap: agents always behave the same way in a third‐party interaction as with the child; that is, behavioral variability between agents is caused by stable, context‐independent traits. This inference may have only been carried out to a limited extent by children in Experiments 1 and 2 (though in the latter, too, children's choice of a better partner increased), but allowed them to select more helpful and skilled partners in Experiments 3 and 4. This conclusion echoes previous findings that young children may require multiple instances of consistent behavior information to generate trait inferences or behavioral predictions, even when the relevant information is explicitly provided to children (Aloise 1993; Boseovski and Lee 2006; Ferguson et al. 1984). The fact that in Experiment 4 children had a stronger preference in Skill compared to Prosociality trials, and prioritized skill in Contrast trials, possibly indicates that children are more willing to expect behavioral consistency in domains that are less dependent on social factors (an idea also supported by the fact that children's preferences for a competent partner did not change across the four test trials); or they may have valued the ability of a partner to provide benefits more than the willingness to do so. Interestingly, Experiment 3 showed that both Hungarian and Japanese children performed better in the second compared to the first block they received, despite the fact that they had to attend to different trait domains. This suggests that children from both cultural contexts—particularly those who initially encountered prosociality differences—generalized from one to the next block, having derived the overhypothesis that kobos' behaviors in the game are likely to be stable.

We also tested samples of adults in Hungary (Experiment 2b) and Japan (Experiment 2c). They chose helpful and skilled partners, implying that they spontaneously interpreted the kobos' behaviors in terms of traits. However, their choices were not at ceiling, so the task was not trivial for them. The result from adult participants is in line with the overall developmental trends we found, suggesting that children at the lower end of our age range (5 years) initially do not show robust preferences, but gradually come to show adult‐like choice behaviors.

When comparing the data from the two different testing contexts, some interesting patterns emerged, which may reflect emerging cultural differences in social cognition and cooperation. First, Japanese children paid relatively more attention to reward differences that were driven by circumstance, rather than agents' behaviors (Experiment 1), which could indicate a higher prioritization of situational factors. Second, both Japanese adults and children showed a stronger preference for skilled agents: They preferred a competent partner more than the corresponding Hungarian samples (Experiments 2b and 2c, and 3a and 3b, respectively). Further, in Contrast trials (Experiment 2), Japanese adults picked a fast, selfish over a helpful, slow partner, unlike Hungarians, who did not prioritize either trait. One possible explanation for this pattern is that instead of invoking trait explanations, Japanese participants interpreted the differences between the kobo in terms of effort. Previous research has found that people from East Asian cultures believe achievements to be determined to a greater extent by effort than ability, while Westerners prefer dispositional explanations (see Dean and Koenig 2019, for a review). In our task, Japanese children and adults may have preferred the agent as a partner who they saw as more hard working. We also found that Japanese children's preference for a more helpful partner was higher than that of Hungarians. A contributing factor to this difference may be that East Asian cultures tend to have a collectivist worldview, while Western cultures emphasize individualism (Nisbett and Masuda 2007). In our study, Japanese children may have been more “group‐minded” and thus more willing to perform the task collaboratively, leading to a greater focus on the prospective partner's qualities. In contrast, Hungarian and Austrian children may have attempted to forage independently rather than rely on help. Note that, since we did not have specific predictions about cultural differences, these findings should be taken as exploratory and require follow‐up investigations. In particular, more diverse samples should be recruited to both assess the generalizability of the results we found, as well as probe potential sources of variability.

Taken together, this set of findings points to the possibility that action understanding, social evaluation, trait attribution, and partner choice may not automatically follow one another, and may rely on distinct cognitive mechanisms. This is in contrast to some theoretical accounts proposing that young infants' preference for helping agents evidences an early‐emerging partner choice psychology which also underlies cooperative decision‐making later in life (e.g., Kuhlmeier et al. 2014; Martin et al. 2019; Van de Vondervoort and Hamlin 2016). Our findings instead speak for a protracted development of trait‐based partner choice and suggest that learning may shape children's categorization and evaluation. First, on the scale of ontogenetic time, children may come to adopt culture‐specific ideas about the existence and taxonomy of character traits. Second, over the course of multiple interactions in a particular context, they may discover that the assumption that agents' behaviors are stable (a phenomenon attributed to traits) serves as a useful principle for selecting advantageous partners. While recent years have seen an increased focus on tracing the development of partner choice psychology across middle childhood (Grueneisen et al. 2023; Hermes et al. 2016; Martin et al. 2022; Prétôt et al. 2020; Woodward et al. 2022), substantial gaps remain in this literature. Existing studies on this topic have generally asked children to perform a hypothetical partner choice where they don't actually interact in a meaningful way with the partner, calling the validity of the choice into question, and/or have presented children with agents' behaviors through verbal vignettes that rely on normatively laden terminology and provide (inadvertent) conceptual scaffolding. The series of experiments reported here aimed to address these shortcomings. Moreover, the structure of the game we developed was such that we could vary both the prosociality and the skill of prospective partners, as well as the interaction of those traits, in the same task. This approach allowed us to assess the respective influence of these two cooperation‐relevant trait domains on children's partner choice.

Still, the present study leaves many questions about the development of trait reasoning and partner choice psychology unanswered, some of which we believe the Co‐Collectors application has the potential to address. Future research could test in a more fine‐grained way the role the trait parameters play in observers' responses, for example, at which trade‐off between prosociality and skill a preference for either emerges. Research could also further probe whether children prioritize traits contingent on the structure of the environment and flexibly adjust their partner choice. Moreover, it could be studied whether children themselves act more prosocially toward certain partners, reciprocate altruistic behavior, or even engage in reputation management in the game.

Overall, our results point to the possibility that there may be distinct mechanisms involved in action understanding, trait attribution, moral evaluation, and partner choice, and that responses children give to a question posed by an experimenter need not reflect their spontaneous social reasoning or behavior in a more realistic context. What is more, studying these issues with tools such as the Co‐Collectors app, which reduce experimenter influence, can facilitate replications across different testing sites and cultural contexts.

## Supporting information


Data S1.

## Data Availability

All materials (stimuli, experimental scripts, data, statistical models, and their output) are available on OSF: https://osf.io/p8uhj/. The analyses presented here (except for those directly comparing results from different samples) were preregistered.
