# Supplementary material for: Children's Trait Inference and Partner Choice in a Cooperative Game
Source: Child Dev. 2025 Apr 12;96(4):1458–73. doi: 10.1111/cdev.14247 (PMC12208008; doi:10.1111/cdev.14247)
Supplement: Supplementary file 1 — Data S1. [file CDEV-96-1458-s001.docx]

Childrens’ trait inference and partner choice in a cooperative game – Supplementary Materials

#

### A: Detailed description of the Co-Collectors game

### Gameplay, agents, and resources

In the Co-collectors game, small, colorful, monster-like autonomous agents called “kobos” collect berries. The berries are contained inside “coconuts”, which fall from a tree and have to be cracked open by the kobos using little hammers (Figure S.1). If a kobo manages to hit a coconut a sufficient number of times, the coconut opens and the berries it holds fly into the kobo’s basket. If, however, the kobo does not deliver enough hits within a certain amount of time, the coconut disappears and its berries are lost.

Kobos can move left and right on the ground in horizontal directions to reach the coconuts, which fall from a large tree at random locations. In each round where there are two kobos, they can move simultaneously and independently from one another without displaying any explicit cues of social interaction.

The coconuts that fall from the tree can be either colorful ones, which match in color a kobo in the scene, or brown ones. Colorful coconuts are “proprietary”: they contain berries of the same color, which are awarded to the matching kobo, irrespective of who cracks them open. In contrast, brown coconuts are “shared”: they contain berries of two different colors, corresponding to the two kobos present in the scene. When the coconut is cracked, the berries inside automatically fly into the basket of the kobo with matching color. The height of the berry piles in the baskets represents the amount of berries collected.

Kobos can crack coconuts alone or jointly. In joint cracking, they deliver hits on opposite sides of the coconut (see Figure S.1). When one of the kobos is operated by a player (always a yellow one), she can move the kobo by dragging it left or right on the screen. The player can crack coconuts by positioning her kobo next to a coconut (at which point a hammer appears) and tapping on it; for each tap, the kobo hits the coconut once with its hammer. At the end of a round, the amount of berries collected by each kobo is displayed in front of their houses. In rounds where the participant herself plays, she can enter into her kobo’s house (by tapping on it), where the berries collected throughout the game accumulate in a pile on the ground.


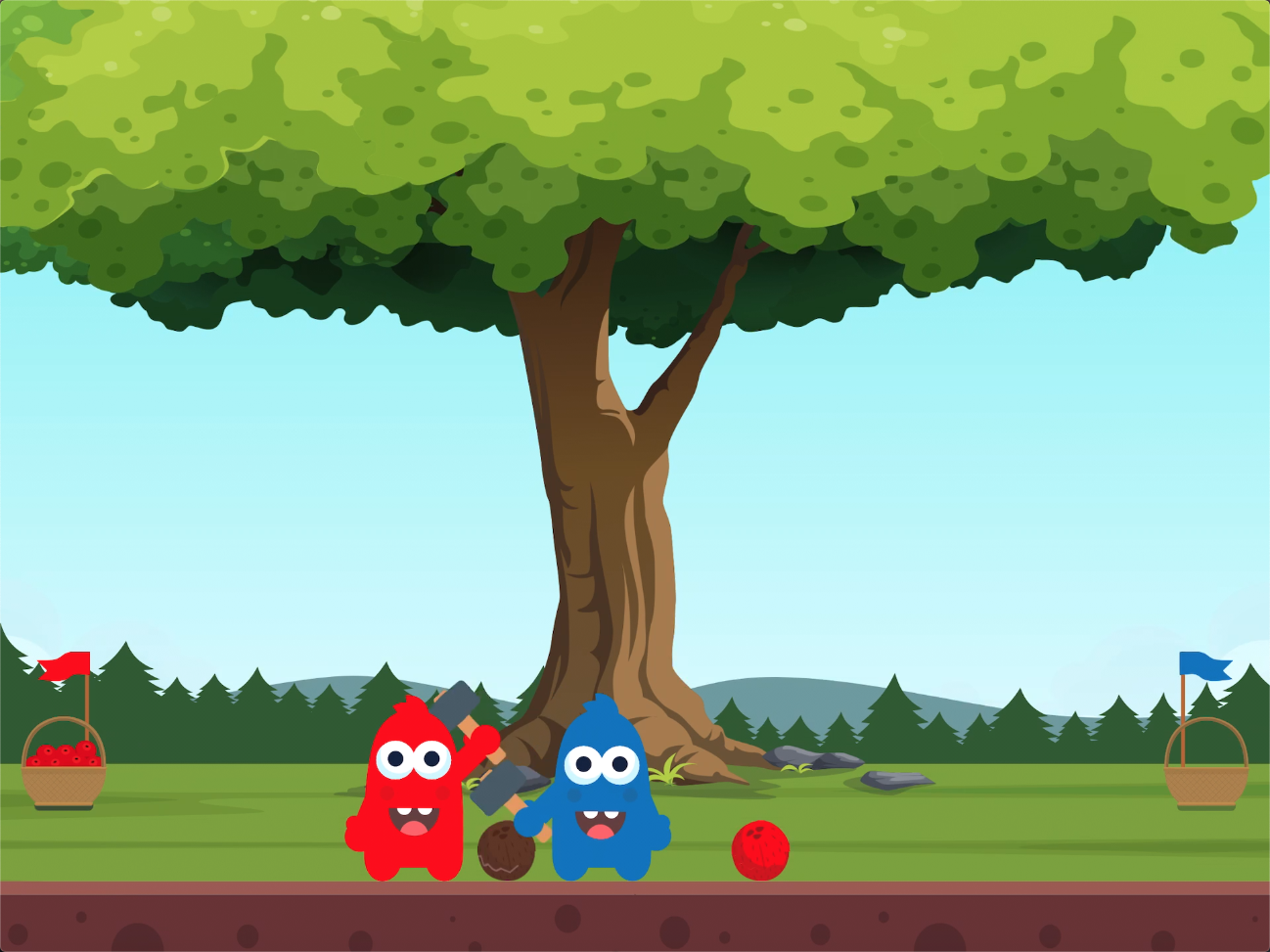


*Figure S.1*. Screenshot of an Observation round.

###

A game is composed of a sequence of rounds specified by the researcher in advance. There are different types of rounds, aimed at introducing children to the game (Practice rounds), allowing them to observe other agents collect berries (Observation rounds, Figure S.1), choose a partner (Partner Choice rounds, Figure S.2), and play together with this partner (Cooperation rounds).

The structure of the game, as well as its specific parameter values (described in the following section), are set through an online configuration interface. During play, the game records the actions of the participant (e.g., partner choices, tapping coconuts) as well as their timing.


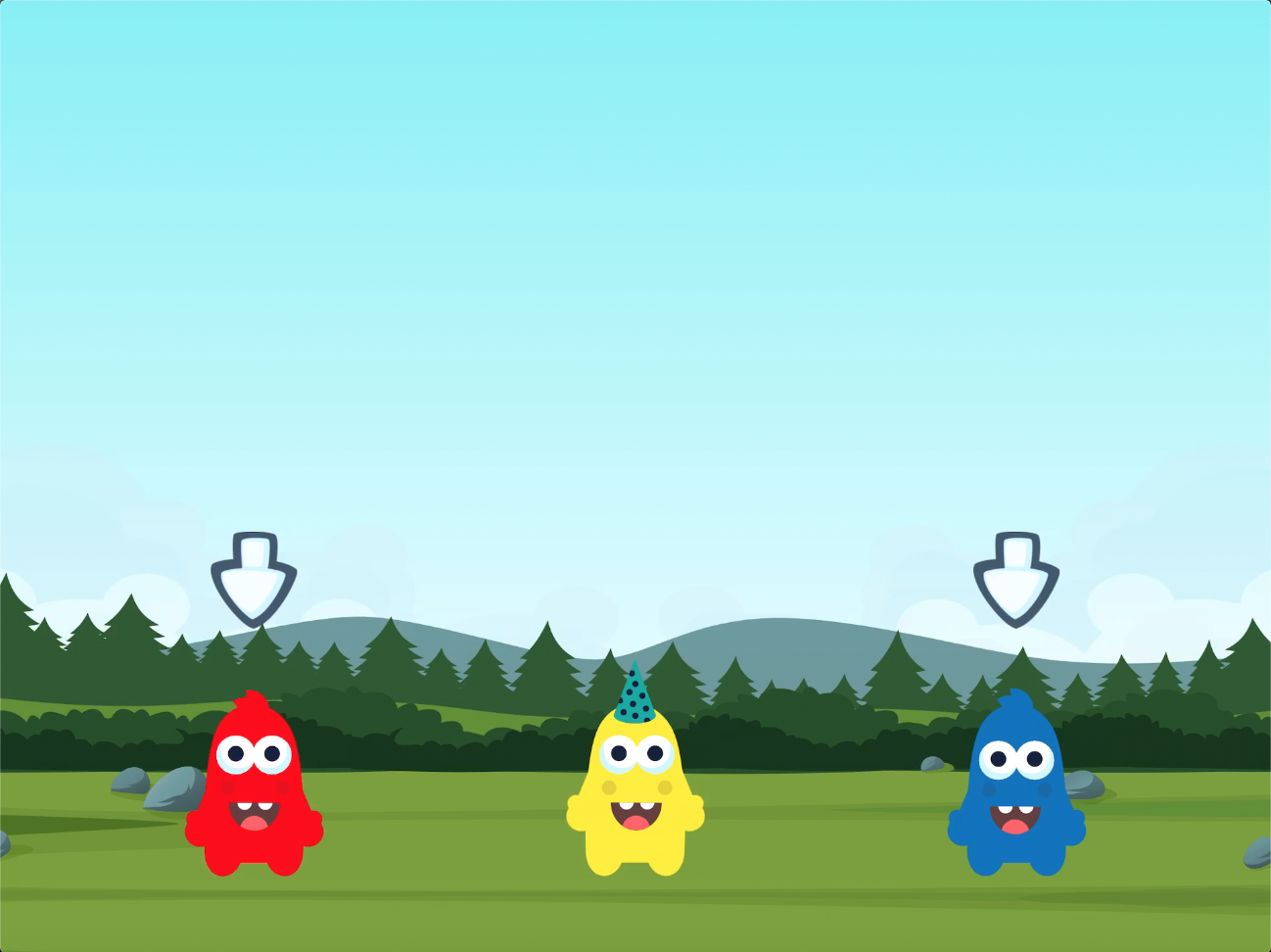


*Figure S.2*. Screenshot of a Partner Choice round.

### Game settings and parameters

The game was designed such that its progression and its parameters for the environment and the agents can be flexibly set by the experimenter. Many of these parameters are stochastic: They are either defined as a value selected randomly from a uniform distribution over a parameter range, or represent the probability of certain events to occur.

The environment parameters define the sets of coconuts available for the players in various rounds. These include the number of coconuts in a round, the probability distribution of coconuts between the two colorful coconuts and the brown coconuts, the coconuts’ hardness (how many hits are required to crack them before they spoil), and the duration that a coconut exists before it spoils.

The agent parameters specify behavioral characteristics along two dimensions: skill and prosociality.

*Skill*. In the context of our game, a highly skilled kobo is one who, all else being equal, is more successful at cracking coconuts and gathering berries. This is implemented by defining the speed at which an agent can approach coconuts, and speed of delivering hits. Because each coconut has internal parameters that specify how much time can elapse until it spoils and how many times it needs to be hit before cracking open, a kobo who is faster is more likely to crack more coconuts and gather berries. To infer that the higher success rate of one kobo reflects its skill, the observer has to assume that this outcome is driven not by random variation in, for example, coconut hardness (such that one kobo fortuitously happens to interact with easily crackable nuts), but that the outcome is caused by its faster response and hit frequency.

*Prosociality*. Social behaviors can be categorized by who pays a cost and who gains in an interaction. In our game, an agent’s prosociality is implemented by a set of parameters that guide which types of coconuts a kobo will likely approach. Importantly, because for the coconut-hardness parameter it is irrelevant whether the hits are delivered by one or two kobos, the chances of successfully cracking a nut tend to be higher when two kobos work together, compared to when a kobo attempts to do so alone. Cracking brown coconuts is a mutualistic behavior (as both kobos receive berries when it is opened), while cracking the partner’s coconut is altruistic (as only the partner receives berries). Conversely, not helping the partner with its coconuts is selfish, and not cracking brown coconuts is spiteful (because the kobo is forgoing a reward for itself in order to prevent the partner from benefiting). An additional cue for the agent’s prosociality is provided by the likelihood with which an agent switches from one coconut to a new one that appears. This is indicative of whose welfare the kobo values *more*, and whether and how much of an opportunity cost a kobo is willing to pay in order to help. Thus, for instance, a very altruistic kobo will abandon its own as well as brown coconuts to help its partner, sacrificing its own berries for the sake of the other. A moderately helpful but ultimately selfish kobo on the other hand will abandon the other’s and the brown coconuts when one of its own color becomes available.

By adjusting the values of the skill and prosociality parameters, one can create profiles for the kobos which specify how they behave in the game. This way, one can systematically vary the target traits between kobos, and flexibly generate Observation and Cooperation rounds that implement the desired kobo personalities. Kobos chosen as partners after an Observation round always have the same personality profile in a subsequent Cooperation round (e.g., a kobo who helped a third party also helps the participant collect resources with the same probabilities).

**B: Average number of rewards obtained by agents in the Observation trials (Experiments 1-4)**

In the following, we provide an overview over how many resources the agents in the Observation round stimuli from our experiments obtained (mean across trials, range).

The number of berries collected by the kobos in Observation trials (i.e., where kobos are computer-animated rather than controlled by the player) depends on the settings of different parameters (e.g., distribution of coconuts of different types, coconut hardness, distance of kobo from a new coconut and time until a coconut spoils), which generate outcomes probabilistically. This means that there is an element of randomness to the events shown in the trial videos. For example, if the distribution of coconuts is set such that brown, "own" and "other" coconuts each appear around 1/3 of the time, a trial with 12 coconuts may feature 4 coconuts of each type, but could also have 3 brown, 5 "own", and 4 "other" coconuts.

The payoffs also depend on the traits and resulting behavior of the agents, specifically, their skill (speed of approaching and hitting coconuts) and helpfulness (probability of approaching different coconut types). In the stimuli we generated, these traits lead to the following outcomes (all else being equal):

(1) If there are proprietary coconuts (and not just brown ones), skilled agents collect more berries than incompetent agents, as they are more successful at cracking coconuts. Both agents are moderately helpful, so the skilled agent will assist the unskilled agent collect some rewards, but ultimately prioritize his own payoff. If there are only brown coconuts, which are shared and contain berries for both agents, both obtain the same amount of rewards.

(2) Selfish agents collect more berries than helpful agents, because the latter forgo their own rewards in order to assist their partner, while selfish agents direct their efforts solely towards collecting berries for themselves.

(3) If the two traits are in contrast with one another (i.e., one agent is skilled and selfish, the other is incompetent and helpful), these effects will be additive such that the difference in payoff between the two agents is larger than if they only differ along one trait dimension.

(4) If both agents are equally skilled and helpful, their payoffs will be approximately the same, unless the distribution of resources is skewed to favor one of them.

In Cooperation rounds, where a participant plays together with a chosen partner, the rewards obtained by both players depends on the behavior of the participant (i.e., how skilled they are, which coconuts they approach). Therefore, we cannot specify how many berries a participant can expect to obtain from playing with the different agents.

**Experiment 1-2 (2 trials each)**

| **Agent** | **Number of berries collected in a round  (mean, range)** |
| --- | --- |
| Skill trials | |
| skilled | 27 (24-30) |
| incompetent | 17 (16-18) |
| Prosociality trials | |
| helpful | 11 (8-14) |
| selfish | 21 (18-24) |
| Contrast trials | |
| skilled, selfish | 22 (18-26) |
| incompetent, helpful | 8 (6-10) |
| Success trials | |
| successful | 33 (32-34) |
| unsuccessful | 19 (18-20) |

**Experiment 3 (4 trials each)**

| **Agent** | **Number of berries collected in a round  (mean, range)** |
| --- | --- |
| Skill trials | |
| skilled | 23.5 (20-26) |
| incompetent | 23.5 (20-26) |
| Prosociality trials | |
| helpful | 11 (8-12) |
| selfish | 31 (24-36) |

**Experiment 4 (4 trials each)**

| **Agent** | **Number of berries collected in a round  (mean, range)** |
| --- | --- |
| Skill trials | |
| skilled | 24 (20-30) |
| incompetent | 19.5 (16-22) |
| Prosociality trials | |
| helpful | 14.5 (12-18) |
| selfish | 25.5 (22-30) |
| Contrast trials | |
| skilled, selfish | 30.5 (28-32) |
| incompetent, helpful | 8 (6-10) |

**C: Additional measure, Experiment 3a**

After the test trials in Experiment 3a, the experimenter asked children a set of questions, which we posed as an exploratory measure of children’s understanding of the game. We showed participants one Skill and Prosociality video each, and asked them (1) who from the video was faster or helped more, respectively, and (2) whether it would be better to play with someone who was faster vs. slower, or helpful vs. selfish. Children were extremely accurate: ((1): 58 of 60 children identified the faster, and 55 of 60 the more helpful agent; (2): 57 of 60 indicated that a faster, and all 60 children that a more helpful kobo partner would be better). These accuracy rates likely in part reflect the fact that the questions were posed at the end of the procedure. Nonetheless, it is possible that the format of the prompt, whereby an experimenter explicitly asked children using trait labels, played a role too.

**D: Additional figures**

**Experiment 1**


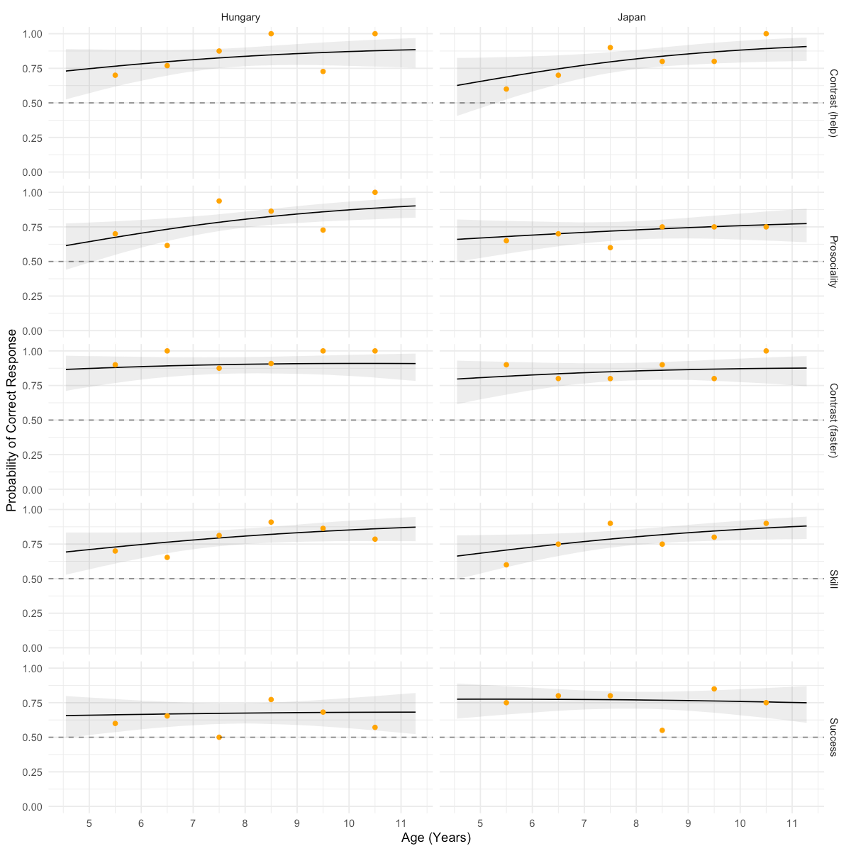


Figure S.3. Age differences in children’s response accuracy in Experiment 1. The proportion of correct responses is indicated by orange dots. The black line indicates the mean of the parameter estimates; the shaded area represents the 89% CI. The left column shows the results for Hungarian (1a), the right for Japanese children (1b). The rows show results for the different trial types, with Contrast trials split by whether children were asked who helped more and who was faster (from the top: Contrast—who helped?; Prosociality; Contrast—who was faster?; Skill; Success).


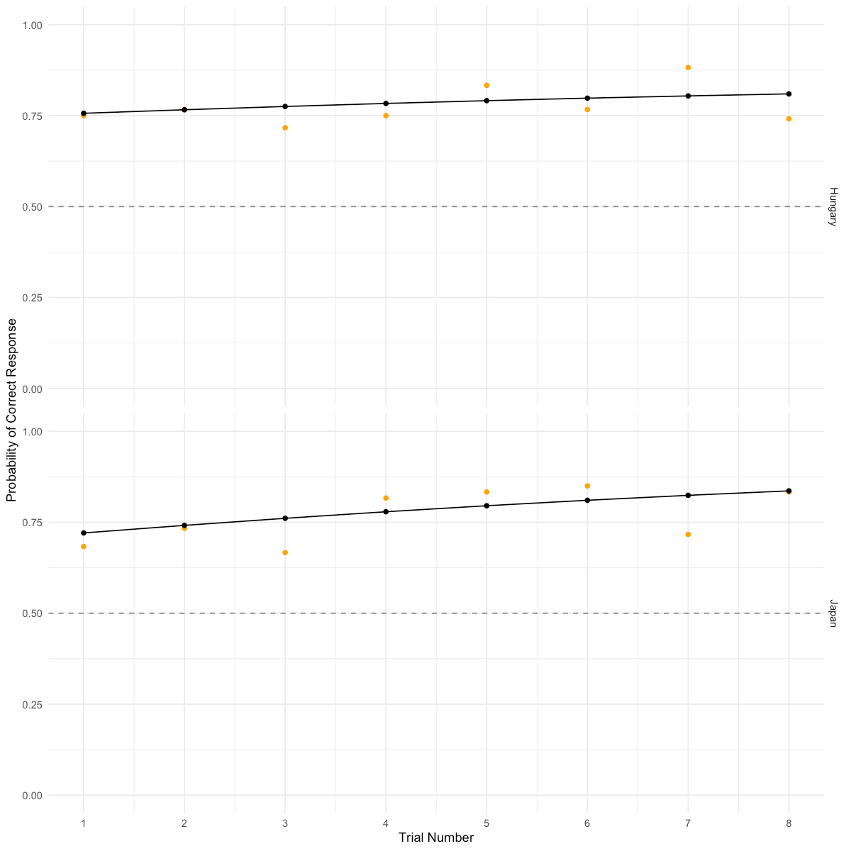


Figure S.4. Children’s average response accuracy as a function of trial position in Experiment 1. The proportion of correct responses is indicated by orange dots. The black dots and line indicate the mean of the parameter estimates. The top row shows the results for Hungarian, the bottom for Japanese children.

**Experiment 2**

**
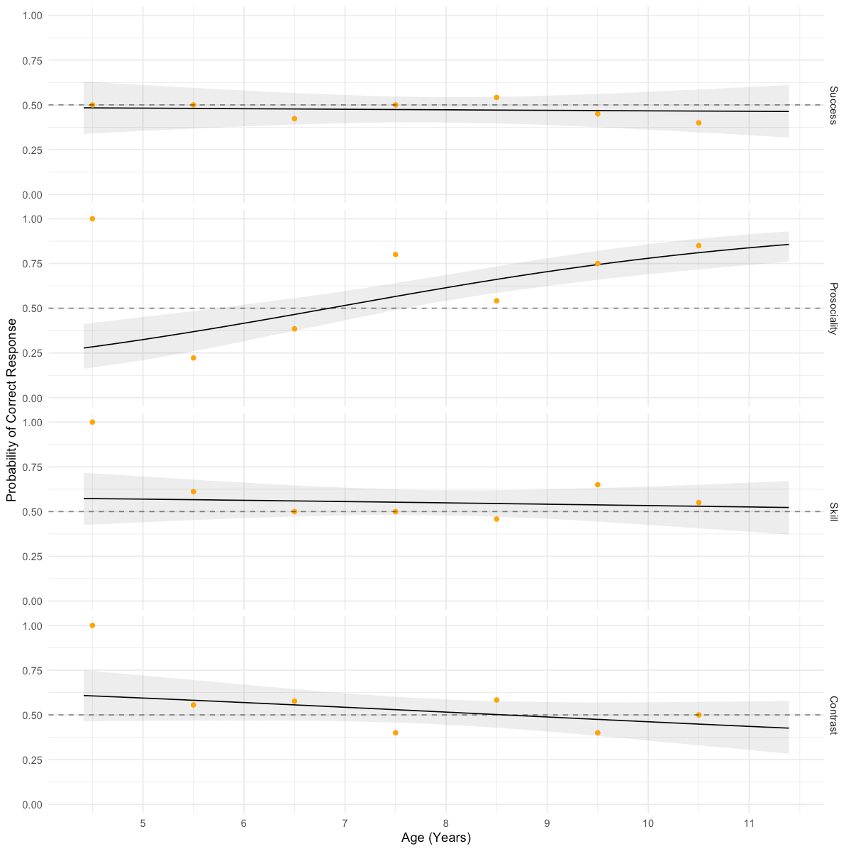
**

Figure S.5. Age differences in children’s response accuracy in Experiment 2a. The proportion of correct responses is indicated by orange dots. The black line indicates the mean of the parameter estimates; the shaded area represents the 89% CI. The rows show results for the different trial types (from the top: Success, Prosociality, Skill, Contrast).

**Experiment 3**

**
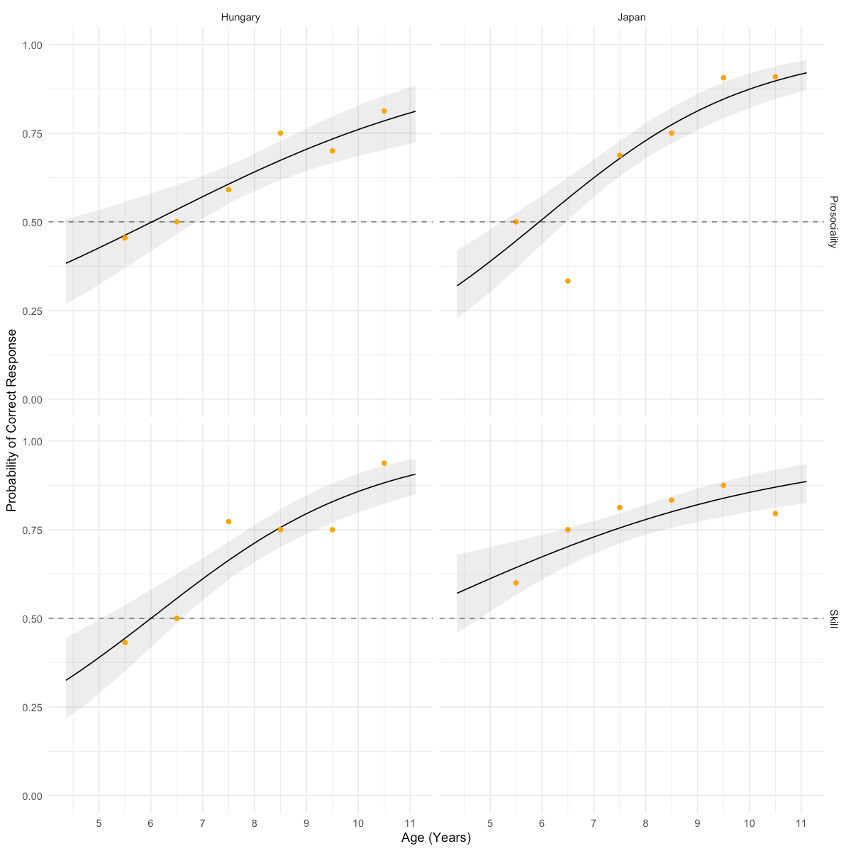
**

Figure S.6. Age differences in children’s response accuracy in Experiment 3. The proportion of correct responses is indicated by orange dots. The black line indicates the mean of the parameter estimates; the shaded area represents the 89% CI. The left column shows the results for Hungarian (Experiment 3a), the right for Japanese children (Experiment 3b). The rows show results for the different trial types (from the top: Prosociality, Skill).


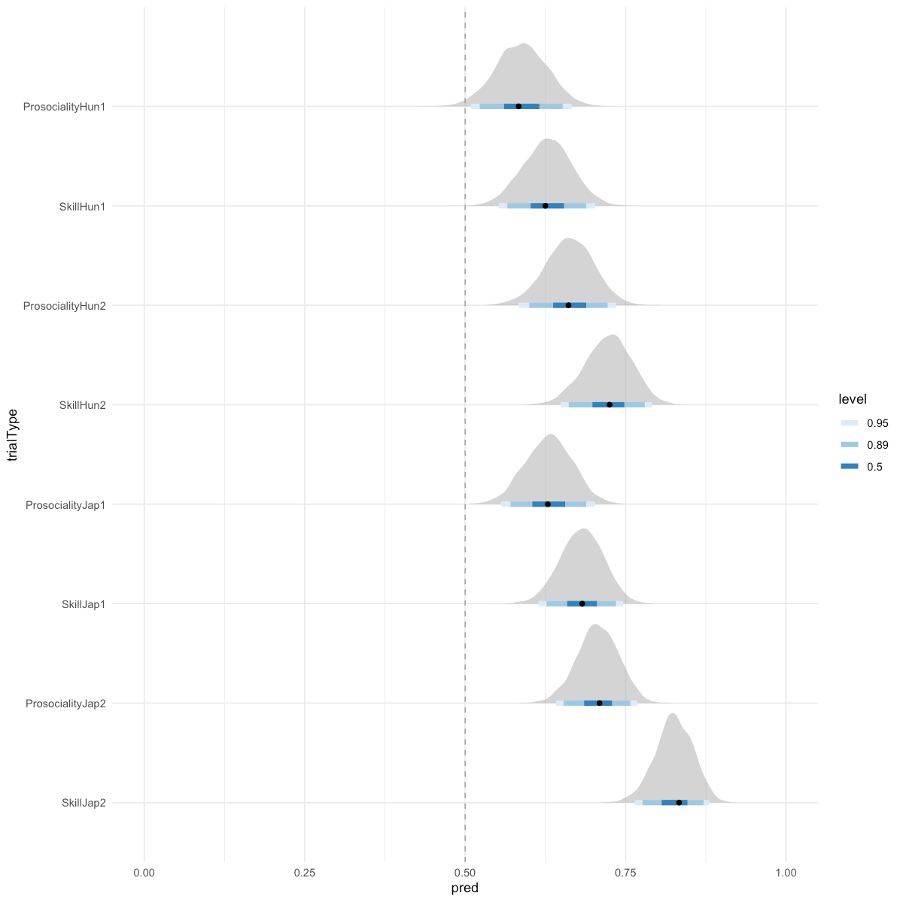


Figure S.7. Children’s response accuracy as a function of Block (i.e., trials were presented in the first or second block) in Experiment 3. The proportion of correct responses is indicated by black dots. The light blue line indicates the 89% CI of parameter estimates, the shaded areas represent the posterior distributions. The rows show results for the different trial types (Prosociality, Skill), split by block (1 or 2) and country.

**Experiment 4**


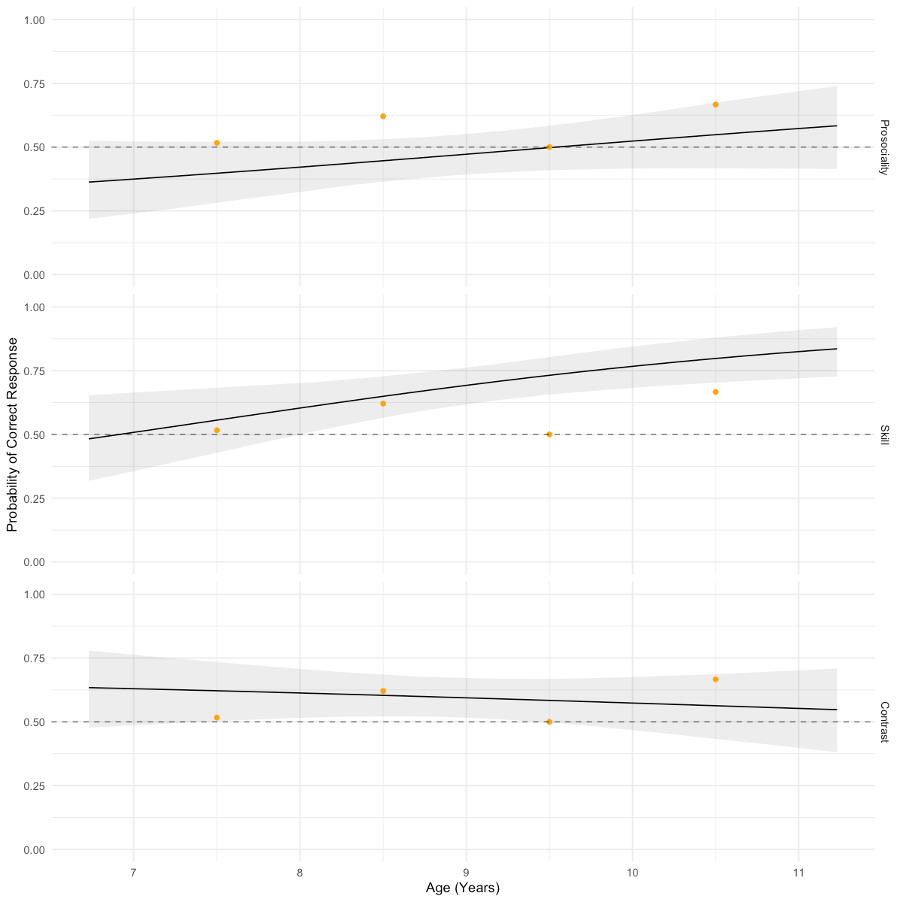


Figure S.8. Age differences in children’s response accuracy in Experiment 4. The proportion of correct responses is indicated by orange dots. The black line indicates the mean of the parameter estimates; the shaded area represents the 89% CI. The rows show results for the different trial types (from the top: Prosociality, Skill, Contrast).


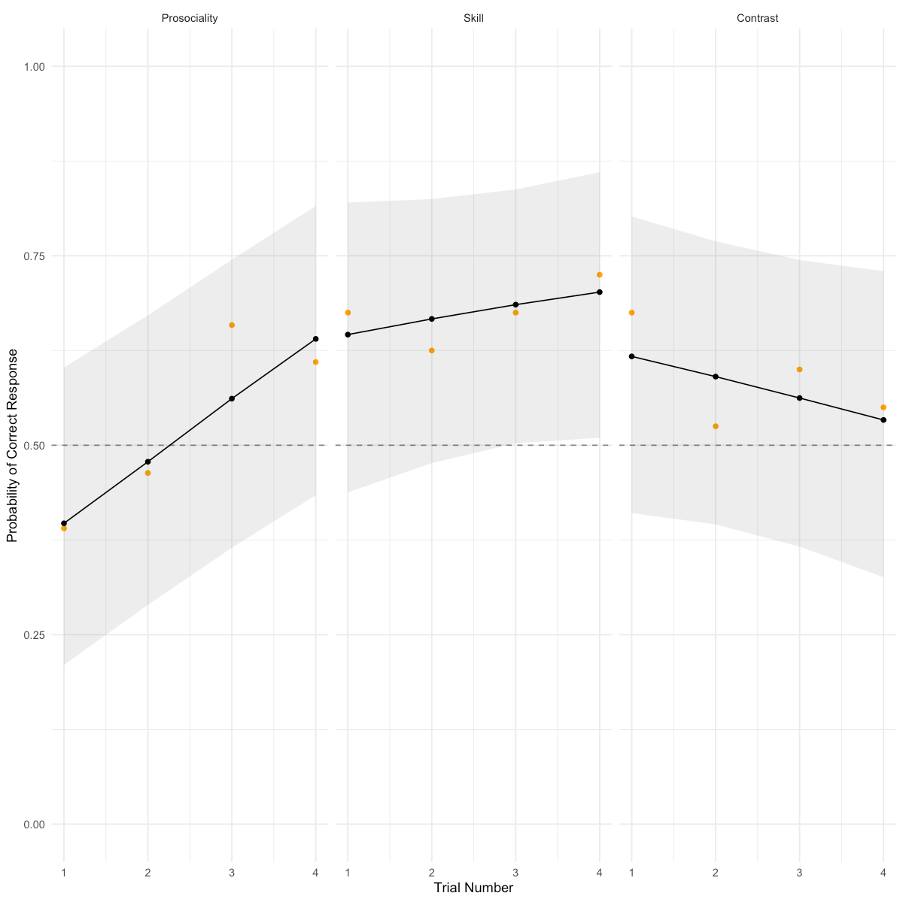


Figure S.9. Children’s response accuracy as a function of Trial position in Experiment 4. The proportion of correct responses is indicated by orange dots. The shaded areas represent the 89% CI. The columns show results for the different conditions/trial types (Prosociality, Skill, Contrast).
